# On the Code of Ethics

**Published:** 1878-11

**Authors:** H. M. Lyman

**Affiliations:** Professor of Pysiology and of Diseases of the Nervous System, Rush Medical College


### ON THE CODE OF ETHICS.

By H. M. Lyman, M. D.

(*Professor of Pysiology and of Diseases of the Nervous System, Rush Medical College.*)

The length of time which has now elapsed since its publication, the high repute in which it has always been held, and the general lack of exact knowledge regarding its requirements, are facts which entitle the Code of Medical Ethics to rank with the sacred writings of the human race. Another proof of the justice of this claim may be found in the growth and gradual attempt at formulation of certain popular beliefs and traditions concerning the document in question. Already we hear of distinctions between the spirit and the letter of the code. Questions of interpretation and matters of doubtful disputation concerning its text, occasionally arise to disturb the quiet of a medical society. All this clearly indicates that the age of comment has arrived. I, therefore, offer the following thoughts in the form of a commentary on the Code of Medical Ethics.

Article I.—*On the duties of physicians to their patients.*

The principal fact worthy of remark in connection with this article is a certain flavor of antiquity—an archaic style of expression—which pleasantly reminds one of the days when the round, red face of the portly old family doctor beamed upon the world over the horizon of a voluminous ruffle, and was kept in good countenance by the splendor of that magic wand which degenerate moderns only know as a gold-headed cane. This peculiarity, however, is not without its value, for it greatly assists the reader in the effort to place before his mind a picture of those happy ages when doctors and patients—especially the patients—shall adopt the code of ethics and regulate their entire existence by its wholesome provisions.

Article II.—*Treats of the obligations of patients to their physicians.*

It is impossible to resist the suspicion that this article was not contained in the original draft of the code. It wears the aspect of an interpolation which must have found its way at a later date into the body of the original text. In no other way can we account for the evident irrelevance of its matter, and its want of logical connection with the remainder of the document. Considered by itself, as a sermon addressed by a venerable laic to an inexperienced youth recently set up in housekeeping, it would rise beyond reproach. But in a paper which is designed for the guidance of professional men, it seems a little out of place to attempt the instruction of people outside of the faculty, over whom the doctor has no authority nor any opportunity for education in accordance with his notions of propriety. It might be urged that the physician may rightly use the article in question as a tract for the instruction and elevation of the laity; but the difficulty in the way of such use lies in the fact that for persons who exhibit a capacity for this kind of training no such advice is needed; and for that vast majority who consider themselves the patrons rather than the clients of a physician, such counsels can only appear provocative to laughter at the expense of any "fussy old fellow who may pretend to tell them when and how they shall call on him for service" I suspect that the entire article was introduced as a concession to certain restive spirits in the drafting convention, who did not exactly relish the wholesale way in which they were being lectured as to their duties, and who could only be appeased by the insertion of something similar for the benefit of outsiders who were not doctors, but who often made their doctors feel as if the administration of a little wholesome advice would be a source of unlimited satisfaction.

On the whole, I think the dignity of the code would be increased by the omission of Article II.

The Second Section of the code treats *of the duties of Physicians to each other, and to the profession at large.*

Of this section, the first article is occupied with *Duties for the support of professional character.* The whole article is full of the most enlightened and liberal counsel for the guidance of the members of our profession; yet, hardly any article in the code is more persistently and consistently disregarded by physicians. How few, comparatively, there are who keep in mind the admonition of even the first paragraph of the article! As Dr. Fothergill wisely remarks, "Each ecclesiastic asserts the dignity of his office, however unworthy he may think some others are to fill it. * * * Another lesson, too, we might learn from them, and that is, the air of respectful gravity they assume at once when speaking of matters theological, and the readiness with which they check any levity of speech on such matters on the part of any one present. * * * It is quite time that we followed the example of the other professions."

There can be no doubt that much of the depreciation of the medical profession in the popular estimation is due to the unbridled license with which even respectable physicians, in conversation with the laity, will seek to undermine the reputation of a rival, and consider themselves perfectly justified in so doing, if that rival chances to belong to some other school of therapeutics than their own. We are too prone to forget that, among the common people, the difference between doctors is not as perceptible as it is among those who have the educational qualifications to judge of such things. For the laity, a doctor is a doctor; and if they hear the members of one wing of the profession speaking evil of the members of another wing, they generally conclude that their speech is based upon jealousy, and draw inferences as unfavorable to the evil speaker as to the evil doer.

The third paragraph of this article treats of the subject of advertising. Nothing can be more admirably conceived, and nothing is more often misconstrued. That this should be true seems rather strange at first sight; but I suspect that much of the misapprehension concerning the true meaning of the provision grows out of indifference to punctuation marks on the part of careless readers. The correct reading of the text is as follows:

"It is derogatory to the dignity of the profession to resort to public advertisements, or private cards, or handbills, inviting the attention of individuals affected with particular diseases—publicly offering advice and medicine to the poor gratis, or promising radical cures," etc.

That is clear enough, but, if I may judge from their behavior, a great many conscientious gentlemen read as if the comma which follows the word *handbills* were a semicolon, or even a full period. This changes the entire meaning of the sentence, and converts all advertising into an offense against the code. On the contrary, the article does not in any way disapprove of the use of "public advertisements, or private cards or handbills;" it only looks with disfavor upon the use of such methods as a means of "inviting the attention of individuals affected with particular diseases—publicly offering advice and medicine to the poor, gratis, or promising radical cures," etc., etc. It follows, therefore, that the well regulated physician who carefully studies his code, and is aware of the unquestionable value of advertising in all business avocations, will proceed to place himself as conspicuously before the public as his brains and purse will permit. In the country villages he will take pains to keep in a prominent column of the local paper, a notice which will inform the public where and when he may be consulted by the sick. He will, very likely, place his business card upon the rack at the tavern, or the railway station, or the post-office. In every way that is consistent with good taste and the customs of the local society, he will seek to make himself known and respected by his fellow citizens of all classes. In the large cities, he will resort to precisely analogous methods; but since it is very expensive to publish a card in the daily papers, he will take pains to advertise in the directory, where his advertisement will be inserted free of expense. In this connection I may remark that the majority of physicians are too indifferent to this matter of advertising in the directory, where for a mere trifle every physician might secure the insertion of his office hours, to his own advantage and to the great additional convenience of the public. There is no ethical reason why the entire contents of the Medical Register should not appear in the City Directory. Very naturally, as the doctor grows prosperous, he will so much the less feel the need of keeping his name before the public through the aid of the press; but this fact should never betray him into any utterance in depreciation of the legitimate efforts of his younger or less fortunate brother.

Were this the place for a dissertation on the art of advertising, I might say much more regarding the matter; but I must leave that to the good sense of each individual, which should suggest numerous methods in perfect harmony with the letter and the spirit of the code. The very fact that certain methods of advertising are disallowed, is sufficient to show that other methods are permitted; and as the forbidden methods are precisely those which the largest experience has always found to be in conflict with the attainment of the highest degree of professional success, it is a logical inference that the code does not in any way discountenance such methods of advertisement as are really conducive to prosperity.

The fourth paragraph is clear, and unobjectionable in its purpose. The prohibition against the use of secret nostrums is, however well meaning the intentions of its authors, a little too stringent; and as a matter of fact it is seldom regarded. Certainly, no one after learing their value was ever deterred from the use of James' Powder, or McMunn's Elixir, or Warburg's Tincture, or J. Colis Browne's Chlorodyne, or Tarrant's Seltzer Aperient, by the consciousness of ignorance regarding their exact composition. Still, as a general rule, the article may pass without a challenge.

The fourth article of this section treats *of the duties of physicians in regard to consultation.*

Of this article, the first paragraph bears evidence of having been carefully framed, in accordance with the euphuistic predilections of the authors, for the purpose of summary dealing with a dark and dismal subject which was too gross for open mention in such a rose-colored document as the Ethical Code. At the time of its composition the medical profession in America was annoyed and disgusted by a pestilent little group of ignorant fellows who were known as homœopathists. A somewhat similar set were called Thompsonians, or botanic doctors. These wretched creatures pretended to have come into possession of new light about the art of healing, and, being generally devoid of knowledge or sound morality, they had no scruples regarding all sorts of reprehensible courses which seemed likely to advance their personal interests.

The consequence was naturally a great outburst of indignation on the part of honorable physicians, and the rapid growth of a general determination to have nothing to do with the accursed thing. Expulsion of the devoted men from the medical societies was at once accomplished. They were denied recognition as physicians, and it was made an unlawful thing for a member of the profession to give them any aid or comfort whatever. The whole proceeding was finally clinched by the introduction into the Code of Ethics of the article now in question.

Time rolled on, and a whole generation has passed away since this decisive action of our ancestors. Here and there exists a survivor of those passionate days, who keeps alive and disseminates the tradition that this article was designed to prevent all consultation with homœopathic and eclectic physicians. Such is the fact, and probably no other article of the code has been more faithfully regarded. Occasionally a hapless brother would transgress, but the eunuchs were always in waiting, and the bowstring or the axe soon made an end of the imprudent offender. All this, however, is now at an end. Rip Van Winkle awakes from his lifelong sleep, and the war of the 'pathies has ceased. Henceforth, it will be hard to find a doctor with whom the most fastidious regular practitioner may not consult.

Strange as it may seem, this great change has unquestionably taken place, and it will be wise for us to recognize the fact, and to shape our course accordingly. Let us see how the matter stands.

The article under consideration declares that: " No intelligent regular practitioner, who has a license to practice from some medical board of known and acknowledged respectability recognized by this (American Medical) Association, and who is in good moral and professional standing in the place in which he resides, should be fastidiously excluded from fellowship, or his aid refused in consultation." *Intelligence*, *regularity*, *license* and *morality* are the four corner-stones upon which rest a doctor's claim to fellowship with the faculty.

About the character of the qualifications covered by the terms *morality* and *intelligence* there is so little question that each individual practitioner can safely be left to judge of their possession by his neighbors. A "*license* to practice from some medical board of known and acknowledged respectability" can now be obtained in many of the States of the Union. The question of *regularity* is the only thing left for consideration. What makes one a "regular practitioner?" Hear the code!

"No one can be considered as a regular practitioner, or a fit associate in consultation, whose practice is based upon an exclusive dogma, to the rejection of the accumulated experience of the profession, and of the aids actually furnished by anatomy, physiology, pathology and organic chemistry."

Thirty years ago, there were men seeking recognition from the public as practitioners of the healing art, to whom this statement would apply, but now the race is almost extinct. A living Dodo or a Moa would hardly excite greater enthusiasm in ornithological circles than would now be aroused among scientific men by the production of a genuine believer in the dogmas of Hahnemann or Thompson.

Since Mahomet would not go to the mountain, the mountain came to Mahomet. Driven into a corner, and forced to rely upon their own resources, these outlaws did what excommunicated heretics have always done. They organized themselves. They attempted to purge themselves of grossness and of palpable ignorance. They went to school. They studied human nature, and sought to win the secret of success. Many of them profess morality. Some are even respectable. They dissect. They walk the hospitals. They are deep in organic chemistry. Their autopsies are as numerous as their opportunities. They read our books, and are sometimes skilled in the use of our own favorite remedies. As for Father Hahnemann—*stat nominis umbra!* He and his beliefs are fast disappearing among the shadows of the past. We reverence Galen and Hippocrates, though we rarely do their works. Let these men canonize whom they will, they are men of to-day and not of the past. In what more then, do such men need for the successful prosecution of a claim to the title of *regularly educated physician* in the meaning of the Code of Ethics? They are physicians—they are educated physicians—they are regular physicians in the sight of the law.

In this interpretation of the article now before us, I can arrogate to myself no priority of thought. It is the expression of a conviction which has long been half concealed in the hearts of progressive men, and the time seems ripe for its utterance. Of course, it is too much to expect that such doctrine will secure unanimous assent. It is too much to expect that the fathers of the profession will consent to meet on equal terms the hoary knave whom they have known since youth as a villain past redemption. Nor is it desirable that such a consummation should be arrived at. Moral character—not scientific opinion, should be the touch-stone of consultation. If by refusing to consult we could deprive every ignoramus of his legal right to practice, the case would be different; but the law has taken from us all such responsibility; consequently, when a man has received his license as a qualified physician, there should be an end of discussion about his recognition as a medical practitioner. But if his moral character is bad, any physician should be at liberty to refuse, on that ground to consult with the offender. This liberty the code already gives—what more do we need for the protection of the much talked of "honor and dignity" of the profession in the matter of consultation?

Did time permit, I might enter at length upon a discussion of the wisdom or unwisdom of the catholicity thus licensed by the code. The subject is now under discussion elsewhere, and many who see that the old barriers have given way are clamorous for new articles which shall restore the former stringency of non-intercourse. But to the intelligent eye it is evident that "old things have passed away," and that a new order has arisen. There will be much lamentation and many wild regrets when it is discovered that Great Pan is really dead, but the true philosopher will rejoice at everything which marks the decline of bigotry and of sectarianism in medicine as well as in religion. Very likely the American Medical Association may be compelled to frame a new article which shall reach the modern "liberal" homœopathist as the old one does not; but the effect of such action will only delay—will not arrest—the inevitable. There are plenty of independent men in the profession who will then boldly say, that the old code is good enough for them, and they will refuse to surrender their new found liberty by reason of any illiberal out-cry in behalf of non-intercourse.

The remaining paragraphs of this article contain nothing that does not commend itself to the good sense of every one. The same thing may be said of the entire contents of Article V. It is only when we reach the second paragraph of Article VI. that anything appears worthy of comment. If this injunction had been always rigidly observed, many of the schisms which have rent the profession, and many of the too willing martyrs who have been offered up in the presence of an unappreciative and scoffing public, would never have been known. It is too late to remedy the scandals of the past; but if it had been possible to follow the examples of other associations, whose discipline and whose punishments are hidden from the public eye, it would have been far better for the reputation of the medical profession. The spirit of this article is utterly opposed to that ecclesiastiform intolerance which has rent us in twain, and has set up a number of jarring sects where all should have joined in one united front for the common good. I hardly need remark that it is, moreover, wholly opposed to such use of the public press, even in the form of a medical journal, as may be calculated to discredit the parties to a difference of opinion about those "points in medical ethics and etiquette through which the feelings of medical men may be painfully assailed in their intercourse with each other."

The concluding section of the code treats *of the duties of the profession to the public, and of the obligations of the public to the profession.*

The first paragraph declares that physicians should "be ever ready to give counsel to the public, in matters especially appertaining to their profession, etc."

This admonition is too often forgotten by the faculty. The medical societies rarely add anything to the knowledge of the public, and the men of learning and experience in the profession almost never break silence for the benefit of any but their own patients. The only attempts to obey this article are made by a few reckless spirits, who, having no reputation to lose, are quite careless about the accuracy of their utterances. Occasionally, there will be a few words of warning or of self glorification from some local sanitary corporation; but, as a general thing, the members of the profession exhibit no desire for the authoritative instruction of the communities in which they dwell.

For this negligence there are several reasons, of which not all are entirely creditable to the faculty. It is easy to see that the doctor who has achieved success by dint of unremitting toil is not very likely to occupy his rare moments of leisure with the painful effort to compose a letter for the columns of the daily paper. Nor is the scholarly young physician any more willing to forego the satisfaction of seeing his work in the current medical journals for the sake of attempting the very difficult task of explaining to an untrained audience matters connected with what must necessarily for such people be an occult science. These difficulties are themselves almost sufficient to deprive the laity of all the advantages which might accrue from the guidance of the best minds in the profession, acting through the medium of that universal school-master—the daily paper. But when to these causes are added the weight of opprobrium which is invariably thrown by the mass of the profession upon anyone who endeavors to obey this article of the code, it is impossible to escape the consequences of such action, and we necessarily find the transient literature of the day almost entirely abandoned to the guidance of the worst representatives of a learned profession. Whence springs this opprobrium which undeniably operates with such force? We are told that it is the proper expression of zeal for "the honor and dignity of the profession" in accordance with the Code of Ethics, which prohibits advertising in the papers. When the inaccuracy of such an assertion is exposed, we are told that the code forbids "inviting the attention of individuals affected with particular diseases;" and it is assumed that a physician cannot appear in print without thus, directly or indirectly, associating himself before the public with some particular disease or class of diseases. Here, then, we find the secret spring of that anxiety about "the honor and dignity of the profession," which is so rampant whenever any accident gives the newspaper-reading portion of the community the smallest ghost of a chance for the inference that one physician may know more than another about certain branches of his calling. It is really not so much a righteous zeal for "the honor and dignity," etc., as it is an expression of professional jealousy.

I do not complain of this, for it is exceedingly characteristic of human nature; but I do complain of calling things by wrong names. It is a fact, beyond question, that the insertion of this clause—"inviting the attention of individuals affected with particular diseases"—is not so much a necessary provision for the honor of the profession, as it is a concession to the weakness of human nature. Men do not like to be reminded of differences which actually exist. I, therefore, find no fault with the restriction. I would not advocate its removal, for, if it were rescinded, and we all should advertise our little pet specialties, we would not one of us be any the better off in the long run. A solitary occupation of the field is the only thing which can give medical advertisements any value.

It is, in this connection, impossible too strongly to deprecate that hyper-ethical feeling which would prohibit men of good judgment and remarkable opportunities for experiment from a public expression of opinion regarding the value of articles which are offered to the public for therapeutical purposes. At present, no one but a quack can utter an opinion which is likely to find its way into the papers without drawing upon himself the fire of all the ethical champions along the line. Consequently, the public is left to the direction of the worst guides possible, or else it must depend upon the published statements of distant authorities, whose value cannot easily be appraised. It is all very well to tell me—a simple tradesman—that Professor Bamberger, of Vienna, recommends the Hunyadi Janos water; but that is not enough. His certificate may be a forgery; I know nothing about the honesty of the man; I want to know what Dr. Allen thinks about it, for he is my neighbor, and I can ask him about the genuineness of his opinion. That is the way the laity think about these things, and it is wrong to deny them this advantage for the sake of gratifying that spirit which, appearing upon the platform with Dennis Kearney, we denounce as communism, but which, when it looks innocently from behind the Code of Medical Ethics, we call *zeal for the honor and dignity of the profession.*

I am well aware thut with many excellent men, the whole discussion is terminated by the assertion that the course which I defend, would be liable to great abuse. Against this objection I would urge the words of Oliver Cromwell: "It will be found an unjust and unwise jealousy to deprive a man of his natural liberty upon the supposition that he might abuse it. When he doth abuse it, judge." If our medical societies were so organized and conducted as to represent the majority and the quality of the faculty, they could easily forestall the private certificates of individual members of the profession, by a proper discharge of their duties to the community through official instructions regarding the character of articles which are introduced to the public notice. But while the present medical chaos is permitted to exist, the opinion of individual experts will be preferred before the deliverances of any local body which has yet its own reputation to win. And if it were the custom thus to pronounce in public upon the quality of articles presented for judgment, the men whose opinion is sought, would be wary enough in the matter of their endorsements, instead of being, as now, liable to fall into the trap of any more than an ordinarily cunning adventurer.

The folowing advertisements appear regularly upon the covers of the most widely circulated English literary magazines :

"A healthy skin and good complexion by using Pears' Transparent Soap. The best for Toilet, Nursery and for Shaving. Recommended in the *Journal of Cutaneous Medicine*, by the Editor, Mr.

ERASMUS WILSON, F. R. S.,

As the most refreshing and agreeable balm for the Skin."

---

"The only Soap for the Complexion. Making the Skin Clear, Smooth and Lustrous.

WRIGHT'S COAL-TAR SOAP.

Highly and extensively recommended for the Toilet and all cases of Cutaneous Disease by Mr. James Startin, M.R.C.S., Surgeon to St. John's Hospital for Disease of the Skin; the late Mr. James Startin, M.D., F.R.C.S., of Saville Row; Mr. McCall Anderson, M.D., F.F.P.S., of Woodside Crescent, Glasgow, and the other leading members of the profession."

Thus interpreted, our code of ethics stands, the embodiment of a wise and liberal policy. Our fathers "builded better than they knew ;" and only jealousy and bigotry can ever mar their work. In the debate which has now begun, may the truly catholic doctrine prevail!

---

Treatment of Un-united Fractures.—(*Med. Press & Circ.*, July 3, 1878.) Mr. Fitzgerald, surgeon to the Melbourne Hospital, in view of the unsuccessful results attained by milder or severer remedies used in treating these cases, strongly advocates the injection of glacial acetic acid, 0.3—0.6, by means of the hypodermic syringe, between the un-united ends of bone. His experience with this method has been uniformly successful. It is attended by sharp pain at first, which quickly subsides. Any cartilaginous thickening that may have been present at first is soon resolved and absorbed, and bony union takes place rapidly, proper position of the fragments being secured by splints.

